# Supplementary material for: Igg Food Antibody Guided Elimination-Rotation Diet Was More Effective than FODMAP Diet and Control Diet in the Treatment of Women with Mixed IBS—Results from an Open Label Study
Source: J Clin Med. 2021 Sep 23;10(19):4317. doi: 10.3390/jcm10194317 (PMC8509634; doi:10.3390/jcm10194317)
Supplement: Supplementary file 1 [file jcm-10-04317-s001.zip › jcm-1296527-supplementary.pdf]

Supplementary Table S1. Presence of specific IgG_{1-4} antibodies in response to 269 foods among women of the group G2-IP (n-21).

Foods	Response level <7.5 µg/ml IgG not elevated	Response level ≥7.5 µg/ml IgG elevated	Response level ≥20.0 µg/ml IgG highly elevated
**VEGETABLES**			
Aubergine	90.5%	9.5%	0.0%
Bamboo shoots	100.0%	0.0%	0.0%
Broad bean	95.2%	4.8%	0.0%
Chard	100.0%	0.0%	0.0%
Broccoli	85.7%	14.3%	0.0%
Rutabaga	95.2%	4.8%	0.0%
Brussels sprout	81.0%	14.3%	4.8%
Beetroot	85.7%	14.3%	0.0%
Onion	90.5%	9.5%	0.0%
Chickpea	90.5%	4.8%	4.8%
Courgette	85.7%	14.3%	0.0%
Pumpkin	100.0%	0.0%	0.0%
Mung bean	100.0%	0.0%	0.0%
Common bean	90.5%	9.5%	0.0%
Pea	90.5%	9.5%	0.0%
Kale	95.2%	4.8%	0.0%
Jute	100.0%	0.0%	0.0%
Cauliflower	100.0%	0.0%	0.0%
Kohlrabi	90.5%	9.5%	0.0%
White cabbage	90.5%	9.5%	0.0%
Red cabbage	76.2%	23.8%	0.0%
Chinese cabbage	100.0%	0.0%	0.0%
Savoy cabbage	100.0%	0.0%	0.0%
Artichoke	100.0%	0.0%	0.0%
Fennel	100.0%	0.0%	0.0%
Carrots	85.7%	14.3%	0.0%
Cucumber	95.2%	4.8%	0.0%
Okra	100.0%	0.0%	0.0%
Olive	95.2%	4.8%	0.0%
Chilli Cayenne	100.0%	0.0%	0.0%
Chilli Habanero	100.0%	0.0%	0.0%
Chilli Jalapeno	100.0%	0.0%	0.0%
Sweet pepper	81.0%	19.0%	0.0%
Parsnip	95.2%	4.8%	0.0%
Tomato	90.5%	9.5%	0.0%
Leek	95.2%	4.8%	0.0%
Radish	90.5%	9.5%	0.0%
Celeriac	85.7%	14.3%	0.0%
Celery	100.0%	0.0%	0.0%
Lentils	95.2%	4.8%	0.0%
Soybean	95.2%	0.0%	4.8%
Asparagus	100.0%	0.0%	0.0%
Spinach	100.0%	0.0%	0.0%
Potatoes	90.5%	9.5%	0.0%
**CEREALS CONTAINING GLUTEN**			
Gluten	23.8%	52.4%	23.8%
Barley	85.7%	14.3%	0.0%
Kamut	90.5%	4.8%	4.8%
Oats	81.0%	9.5%	9.5%
Wheat	52.4%	33.3%	14.3%
Spelt	61.9%	28.6%	9.5%
Rye	61.9%	23.8%	14.3%
**GLUTEN-FREE CEREALS AND ALTERNATIVES**			
Amaranth	100.0%	0.0%	0.0%
Buckwheat	90.5%	4.8%	4.8%

Carob (St John's bread)	100.0%	0.0%	0.0%
Chestnut	95.2%	4.8%	0.0%
Quinoa	95.2%	0.0%	4.8%
Maize	100.0%	0.0%	0.0%
Lupine	95.2%	4.8%	0.0%
Cassava	100.0%	0.0%	0.0%
Arrowroot	100.0%	0.0%	0.0%
Teff	100.0%	0.0%	0.0%
Fonio	100.0%	0.0%	0.0%
Millet	100.0%	0.0%	0.0%
Rice	95.2%	4.8%	0.0%
Sweet potato	95.2%	4.8%	0.0%
Jerusalem artichoke	100.0%	0.0%	0.0%
Tapioca	100.0%	0.0%	0.0%
**ALGAE**			
Red algae (nori)	81.0%	14.3%	4.8%
Spirulina	71.4%	28.6%	0.0%
**FRUIT**			
Gooseberry	100.0%	0.0%	0.0%
Ananas	85.7%	14.3%	0.0%
Watermelon	85.7%	14.3%	0.0%
Avocado	100.0%	0.0%	0.0%
Banana	85.7%	9.5%	4.8%
Lingonberry	95.2%	4.8%	0.0%
Blueberry	95.2%	4.8%	0.0%
Peach	95.2%	4.8%	0.0%
Lemon	95.2%	4.8%	0.0%
Date	100.0%	0.0%	0.0%
Fig	100.0%	0.0%	0.0%
Pomegranate	100.0%	0.0%	0.0%
Grapefruit	100.0%	0.0%	0.0%
Pear	95.2%	4.8%	0.0%
Guava	100.0%	0.0%	0.0%
Apple	100.0%	0.0%	0.0%
Blackberry	100.0%	0.0%	0.0%
Kiwi	81.0%	14.3%	4.8%
Lychee	100.0%	0.0%	0.0%
Lime	90.5%	9.5%	0.0%
Raspberry	100.0%	0.0%	0.0%
Tangerine	90.5%	9.5%	0.0%
Mango	95.2%	4.8%	0.0%
Honeydew melon	100.0%	0.0%	0.0%
Yellow plum	85.7%	9.5%	4.8%
Apricot	100.0%	0.0%	0.0%
Nectarine	85.7%	9.5%	4.8%
Prickly pear	95.2%	4.8%	0.0%
Papaya	100.0%	0.0%	0.0%
Quince	100.0%	0.0%	0.0%
Orange	90.5%	4.8%	4.8%
Currants (red and black mixed)	100.0%	0.0%	0.0%
Rhubarb	100.0%	0.0%	0.0%
Sea buckthorn	100.0%	0.0%	0.0%
Plum	90.5%	9.5%	0.0%
Strawberry	90.5%	4.8%	4.8%
Grape	100.0%	0.0%	0.0%
Cherry	85.7%	14.3%	0.0%
Cranberry	95.2%	4.8%	0.0%
**YEAST**			
Yeast	71.4%	23.8%	4.8%
**SPICES**			
Aniseed	95.2%	4.8%	0.0%

Basil	100.0%	0.0%	0.0%
Horseradish	90.5%	9.5%	0.0%
Cinnamon	100.0%	0.0%	0.0%
Savory	100.0%	0.0%	0.0%
Garlic	90.5%	4.8%	4.8%
Wild garlic	100.0%	0.0%	0.0%
Nutmeg	100.0%	0.0%	0.0%
Mustard seed	95.2%	4.8%	0.0%
Clove	100.0%	0.0%	0.0%
Ginger	85.7%	14.3%	0.0%
Juniper berry	100.0%	0.0%	0.0%
Capers	100.0%	0.0%	0.0%
Cardamom	100.0%	0.0%	0.0%
Caraway	100.0%	0.0%	0.0%
Coriander	100.0%	0.0%	0.0%
Dill	100.0%	0.0%	0.0%
Cumin	100.0%	0.0%	0.0%
Lavender	100.0%	0.0%	0.0%
Bay leaf	100.0%	0.0%	0.0%
Lovage	100.0%	0.0%	0.0%
Alfalfa	95.2%	4.8%	0.0%
Marjoram	100.0%	0.0%	0.0%
Lemon balm	100.0%	0.0%	0.0%
Oregano	100.0%	0.0%	0.0%
Paprika, spice	95.2%	4.8%	0.0%
Pepper, white	100.0%	0.0%	0.0%
Pepper, black	100.0%	0.0%	0.0%
Parsley	100.0%	0.0%	0.0%
Rosemary	100.0%	0.0%	0.0%
Cress	100.0%	0.0%	0.0%
Saffron	100.0%	0.0%	0.0%
Salvia	100.0%	0.0%	0.0%
Chive	100.0%	0.0%	0.0%
Chervil	100.0%	0.0%	0.0%
Thyme	100.0%	0.0%	0.0%
Vanilla	81.0%	14.3%	4.8%
Allspice	100.0%	0.0%	0.0%
	**FISH**		
Anchois	95.2%	4.8%	0.0%
Patagonian toothfish	100.0%	0.0%	0.0%
Pollock	85.7%	14.3%	0.0%
Gilthead	100.0%	0.0%	0.0%
Codfish	100.0%	0.0%	0.0%
Plaice	95.2%	4.8%	0.0%
Halibut	95.2%	4.8%	0.0%
Lobster	100.0%	0.0%	0.0%
Squid	100.0%	0.0%	0.0%
Redfish	85.7%	9.5%	4.8%
Carp	100.0%	0.0%	0.0%
Shrimp	95.2%	4.8%	0.0%
Salmon	100.0%	0.0%	0.0%
Snapper	100.0%	0.0%	0.0%
Mackerel	100.0%	0.0%	0.0%
Swordfish	100.0%	0.0%	0.0%
Sea bass	95.2%	4.8%	0.0%
Blue mussels	100.0%	0.0%	0.0%
Octopus	100.0%	0.0%	0.0%
Oyster	85.7%	14.3%	0.0%
Panga (iridescent shark)	85.7%	14.3%	0.0%
Haddock	100.0%	0.0%	0.0%
Scallop	100.0%	0.0%	0.0%

Trout	90.5%	9.5%	0.0%
Crayfish	100.0%	0.0%	0.0%
Shark	100.0%	0.0%	0.0%
Zander	100.0%	0.0%	0.0%
Sardine	95.2%	4.8%	0.0%
Herring	90.5%	9.5%	0.0%
Sole	100.0%	0.0%	0.0%
Tuna	95.2%	4.8%	0.0%
Eel	95.2%	4.8%	0.0%
Angler	95.2%	4.8%	0.0%
**MUSHROOM**			
Oyster mushrooms	100.0%	0.0%	0.0%
Cep (boletus)	100.0%	0.0%	0.0%
Shiitake	100.0%	0.0%	0.0%
Meadow mushrooms	100.0%	0.0%	0.0%
Chanterelle	100.0%	0.0%	0.0%
Bay boletus	100.0%	0.0%	0.0%
**MEAT**			
Veal	95.2%	4.8%	0.0%
Wild boar	95.2%	4.8%	0.0%
Goose	100.0%	0.0%	0.0%
Turkey	100.0%	0.0%	0.0%
Lamb	100.0%	0.0%	0.0%
Deer	100.0%	0.0%	0.0%
Duck	100.0%	0.0%	0.0%
Rabbit	95.2%	4.8%	0.0%
Chicken	95.2%	4.8%	0.0%
Goat meat	100.0%	0.0%	0.0%
Ostrich meat	100.0%	0.0%	0.0%
Quail	100.0%	0.0%	0.0%
Roe deer	100.0%	0.0%	0.0%
Pork	90.5%	4.8%	4.8%
Beef	90.5%	9.5%	0.0%
Hare	95.2%	4.8%	0.0%
**SEEDS AND NUTS**			
Cacao bean	100.0%	0.0%	0.0%
Coconut	95.2%	0.0%	4.8%
Poppy seed	90.5%	4.8%	4.8%
Almond	95.2%	0.0%	4.8%
Sunflower seeds	90.5%	4.8%	4.8%
Cashews	95.2%	0.0%	4.8%
Brazil nuts	90.5%	4.8%	4.8%
Hazelnuts	95.2%	0.0%	4.8%
Macadamia nuts	95.2%	0.0%	4.8%
Walnut	100.0%	0.0%	0.0%
Pine nuts	100.0%	0.0%	0.0%
Peanuts	90.5%	9.5%	0.0%
Pumpkin seeds	95.2%	0.0%	4.8%
Pistachio nuts	90.5%	4.8%	4.8%
Sesame seeds	95.2%	4.8%	0.0%
Flax, linseed	71.4%	23.8%	4.8%
**EGGS**			
Chicken egg white	23.8%	23.8%	52.4%
Geese egg	57.1%	28.6%	14.3%
Quail egg	38.1%	47.6%	14.3%
Chicken egg yolk	38.1%	42.9%	19.0%
**ADDITIVES**			
Agar (E406)	81.0%	19.0%	0.0%
Aloe	100.0%	0.0%	0.0%
Aspergillus niger	90.5%	9.5%	0.0%
Sodium benzoate (E211)	100.0%	0.0%	0.0%

Guar gum (E412)	66.7%	28.6%	4.8%
Xantan gum (E415)	90.5%	4.8%	4.8%
Candied lemon zest	100.0%	0.0%	0.0%
Carrageen (E407)	100.0%	0.0%	0.0%
Curcumin (E100)	95.2%	4.8%	0.0%
Sorbic acid (E200)	100.0%	0.0%	0.0%
Vine leaves	100.0%	0.0%	0.0%
Pectins (E440)	95.2%	4.8%	0.0%
Tragacanth (E413)	100.0%	0.0%	0.0%
**MILK PRODUCTS**			
Kefir	57.1%	23.8%	19.0%
Mare’s milk	90.5%	9.5%	0.0%
Goat milk, goat cheese	76.2%	14.3%	9.5%
Cow's milk	28.6%	33.3%	38.1%
Cooked cow's milk	57.1%	23.8%	19.0%
Sheep's milk, sheep's cheese	85.7%	9.5%	4.8%
Camel’s milk	95.2%	4.8%	0.0%
Sour-milk products	38.1%	33.3%	28.6%
Halloumi	85.7%	14.3%	0.0%
Cow’s rennet cheese	85.7%	14.3%	0.0%
Ricotta cheese	47.6%	28.6%	23.8%
**LETTUCES**			
Chicory	95.2%	4.8%	0.0%
Endive	100.0%	0.0%	0.0%
Radicchio	95.2%	4.8%	0.0%
Dandelion	100.0%	0.0%	0.0%
Lamb’s lettuce	90.5%	9.5%	0.0%
Arugula	100.0%	0.0%	0.0%
Iceberg lettuce	95.2%	4.8%	0.0%
Iceberg lettuce	100.0%	0.0%	0.0%
Lollo rosso	100.0%	0.0%	0.0%
Romaine lettuce	100.0%	0.0%	0.0%
**COFFEE, TEA**			
Rose hip	100.0%	0.0%	0.0%
Rooibos tea	100.0%	0.0%	0.0%
Tea, black	100.0%	0.0%	0.0%
Tea, green	100.0%	0.0%	0.0%
Coffee	100.0%	0.0%	0.0%
Peppermint	95.2%	4.8%	0.0%
Nettle	100.0%	0.0%	0.0%
Camomile	100.0%	0.0%	0.0%
Tannin	100.0%	0.0%	0.0%
**SWEETENERS**			
Cane sugar	100.0%	0.0%	0.0%
Honey (mixed)	76.2%	14.3%	9.5%
Maple syrup	100.0%	0.0%	0.0%
Agave nectar	85.7%	14.3%	0.0%
